# Searching for the Bacterial Effector: The Example of the Multi-Skilled Commensal Bacterium *Faecalibacterium prausnitzii*

**DOI:** 10.3389/fmicb.2018.00346

**Published:** 2018-03-06

**Authors:** Rebeca Martín, Luis G. Bermúdez-Humarán, Philippe Langella

**Affiliations:** National Institute of Agricultural Research, Commensals and Probiotics-Host Interactions Laboratory, Micalis Institute, AgroParisTech, Paris-Sud University, Jouy-en-Josas, France

**Keywords:** probiotic, commensal, *Faecalibacterium*, bacterial effectors, live-biotherapeutics

## Abstract

*Faecalibacterium prausnitzii* represents approximately 5% of the total fecal microbiota in healthy adults being one of the most abundant bacterium in the human intestinal microbiota of healthy adults. Furthermore, this bacterium has been proposed to be a sensor and a major actor of the human intestinal health because of its importance in the gut ecosystem. In this context, *F. prausnitzii* population levels have been found to be reduced in patients suffering from several syndromes and diseases such as inflammatory bowel diseases. These diseases are characterized by a breakage of the intestinal homeostasis called dysbiosis and the use of *F. prausnitzii* as a next generation probiotic (also called live biotherapeutics) has been proposed as a natural tool to restore such dysbiosis within the gut. Nevertheless, despite the potential importance of this bacterium in human health, little is known about its main effectors underlying its beneficial effects. In this perspective note, we aim to present the actual state in the research about *F. prausnitzii* effectors and the future milestones in this field.

## Introduction

Nowadays, humans can be considered as “meta-organisms” composed of 10-fold more microorganisms than human cells (Neish, 2009), which means 150-fold more genes than the human genome itself (Qin et al., 2010; Bruls and Weissenbach, 2011). These microorganisms, named microbiota (and by extension all their genes as a whole, named microbiome) are different depending on the tissue considered. The human gastrointestinal tract (GIT) is one of the most complex ecosystems. The advances of molecular techniques have shown that the collective adult human GIT microbiota is composed of up to 1000–1150 bacterial species (Frank et al., 2007; Qin et al., 2010). The predominant species (46–58%) are those with low GC-content being the clostridium group the most abundant (Zoetendal et al., 2002; Qin et al., 2010). As a consequence of the mutualism established between the host and its microbiota, the GIT micro-ecosystem is key to maintain the homeostasis of a healthy individual (Leser and Molbak, 2009). Indeed, gut microbiota supplies essential nutrients, metabolize indigestible compounds and protects the host against colonization by opportunistic pathogens (Leser and Molbak, 2009; Martín et al., 2013). It also contributes to the development of the intestinal architecture as well as several immuno-modulatory functions (Mazmanian et al., 2005). In some abnormal conditions, an imbalance in the microbial ecosystem may happen leading to a microbial imbalance known as dysbiosis. This dysbiosis is characterized by the growth of different non-predominant bacteria and/or the depletion of commensal ones that can lead to a situation of illness. As a result, this imbalance can also lead to the lack of some beneficial effects of these commensal bacteria, and thus unchain pathogenic conditions not only due to pathogen overgrowth (Martín et al., 2013).

## The Human Gut Microbiota as a Source of Next Generation Probiotics (NGPs)

As gut microbiota is now considered as a major actor underlying health, the idea of using some well-known microbiota components as next generation probiotics (NGPs) is very promising. Probiotics are “live microorganisms which when administered in adequate amounts confer a health benefit on the host” (FAO/WHO, 2001). Recently, this definition has been clarified by an expert panel of the International Scientific Association for Probiotics and Prebiotics (ISAPP) and re-defined as: “live microorganisms that, when administrated in adequate amounts, confer a health benefit on the host” (Hill et al., 2014). Most of the traditional probiotics belong to both lactic acid bacteria (LAB) and *Bifidobacteria* groups. These novel probiotics are thus considered as NGPs in contrast to traditional ones, which were not selected on the basis of human microbiota analysis. To consider a strain as probiotic, it should be: (i) well-characterized, (ii) achieve safety requirements, and (iii) confer beneficial effects on the host. However, a careful selection should be made in each case as probiotic properties are usually strain specific and cannot be extrapolated to another strain even belonging to the same species (Pineiro and Stanton, 2007; Gareau et al., 2010).

Similarly, as probiotic therapy is mainly based on restoring the normal balance of the intestinal ecosystem, we consider that the use of commensal bacteria as NGPs is a natural way to restore the dysbiotic situation within the GIT (Miquel et al., 2015a). Nevertheless, in contrast to most of probiotic lactobacilli or bifidobacteria strains, these NGPs are not listed neither on QPS (for Qualified Presumption of Safety), nor on GRAS (for Generally Recognized As Safe organisms) lists. Furthermore, as they have not a long record of safe consumption (precisely no documented safe use in Europe prior to 1997), these NGPs can only be used either as novel foods or drugs and the requirements to allow their market in Europe are more severe than for conventional probiotic strains (Miquel et al., 2015a). Nowadays, the inclusion of *Faecalibacterium* on the QPS list might be difficult due to its lack of a history of safe use. In addition, full toxicology assays and characterization of the strain are still needed for regulatory approval (Brodmann et al., 2017). We have reviewed this problematic in detail elsewhere (Miquel et al., 2015a).

## A Focus on *Faecalibacterium praustnizii*

As *Faecalibacterium prausnitzii* is an extremely oxygen sensitive (EOS) bacterium and difficult to grow (Duncan et al., 2002), most of the data about its physiology are based on metagenomic studies (Miquel et al., 2013), with some exceptions (Duncan et al., 2002; Ramirez-Farias et al., 2009; Lopez-Siles et al., 2012; Foditsch et al., 2014; Martín et al., 2017). *F. prausnitzii* is a member of the Clostridium group (phylum Firmicutes, class Clostridia, family Ruminococcaceae), specifically of the *C. leptum* group (Benevides et al., 2017), and represents around 5% of the total fecal microbiota in healthy adults being one of the most abundant bacterium in the human intestinal microbiota in healthy conditions (Hold et al., 2003). The first isolates were classified as *Fusobacterium praustnizzi*, but latter on its close relation with members of the *C. leptum* group was established thorough analysis of 16S rRNA gene (Miquel et al., 2014). The establishment of *F. prausnitzii* along the GIT may result from a combination of environmental factors such as other commensal species, redox mediators, oxygen concentration, mucus layer as well as bile salt concentrations and pH (Duncan et al., 2009; Lopez-Siles et al., 2012). In early infancy, *F. prausnitzii* abundance is very low and increases after the establishment of primo-colonizing bacteria (Hopkins et al., 2005). In the last years, this bacterium has been suggested as a biosensor and a major actor of the human intestinal health (Miquel et al., 2013). Indeed, *F. prausnitzii* levels have been found to be reduced in patients suffering from several syndromes and diseases such as inflammatory bowel diseases (IBDs), irritable bowel syndrome (IBS), colorectal cancer (CRC), obesity, and celiac disease (Balamurugan et al., 2008; Sokol et al., 2008; Neish, 2009; De Palma et al., 2010; Furet et al., 2010; Rajilic-Stojanovic et al., 2011) as well as in frail elderly (van Tongeren et al., 2005). We have more deeply review *F. prausnitzii* physiology and beneficial effect elsewhere (Miquel et al., 2013, 2014). Nevertheless, its EOS condition, make viable intestinal delivery one of the current challenges, due to their stringent survival conditions (El Hage et al., 2017).

Because of its important role in GIT homeostasis, *F. prausnitzii* is considered today as a potential NGPs. Its potential utilization has been already proposed for livestock animals, for instance the isolation and characterization of *F. prausnitzii* strains from stool of calves and piglets have been already performed (Foditsch et al., 2014). Also a specific formulation keeping this EOS bacterium alive at ambient air conditions has been also proposed for patients with intestinal dysbiosis-associated diseases (Khan et al., 2014). In order to evaluate its potential beneficial effects as a NGP, we have successfully used this bacterium in several murine models of IBD and IBS (Sokol et al., 2008; Martín et al., 2014a; Laval et al., 2015; Miquel et al., 2016) and other groups have also clearly demonstrated its beneficial effects *in vivo* (Carlsson et al., 2013; Rossi et al., 2015, 2016). Briefly, we have found that mice treated with either *F. prausnitzii* A2-165 or its supernatant (SN) present lower symptoms of inflammation in both acute and chronic chemically-induced colitis models as well as improved gut permeability and function in a model of gut impairment induced by dinitrobenzene sulfonic acid (DNBS) (Sokol et al., 2008; Martín et al., 2014a, 2015; Laval et al., 2015). We have also observed that A2-165 strain was able to reduce pain sensibility in partial restraint stress (PRS) and neo-maternal separation (NMS) murine models (Miquel et al., 2016). Furthermore, Carlsson et al. (2013) and Rossi et al. (2015, 2016) have found similar anti-inflammatory results as well as restoration of increased intestinal permeability in dextran sulfate sodium (DSS) induced colitis.

Staring the host pathways involved in the beneficial effects displayed by *F. prausnitzii*, *in vitro* tests have shown that: (i) although *F. prausnitzii* itself had no effect on IL-1β-induced NF-κB activity, its SN abolished it in Caco-2 cells transfected with a reporter gene for NF-κB activity and (ii) peripheral blood mononuclear cell (PBMC) stimulation by *F. prausnitzii* led to significantly lower IL-12 and IFN-γ production levels and higher secretion of IL-10 (Sokol et al., 2008). In this sense, human dendritic cells (DCs) stimulation with A2-165 and HTF-F strains also induced the production of IL-10 (Rossi et al., 2015). IL-10 has been established to be an important immune-regulatory cytokine that successfully suppresses the exacerbated mucosal immune response associated with colonic inflammation (Schreiber et al., 2000). This increasing in IL-10 induced by *F. praustnizii* has also been observed *in vivo* (Sokol et al., 2008). Furthermore, Rossi et al. (2016) found that the strain of *F. prausnitzii* A2-165 has a strong capacity to induce IL-10 in human and murine DCs and influence the T-cell differentiation *in vitro* and *in vivo*. In this sense, *F. prausnitzii* is also able to increase lymphocyte T regulatory (Treg) population *in vivo* after a colonic chemical challenge (Martín et al., 2014a) and has been identified as the major inducer of a specific IL-10 secreting Treg subset named CD4CD8α lymphocytes present in the human colonic lamina propria and blood which is deficient in IBD patients (Sarrabayrouse et al., 2014).

## *Faecalibacterium praustnizii* Effectors: Where Do We Stand?

**FIGURE 1:**
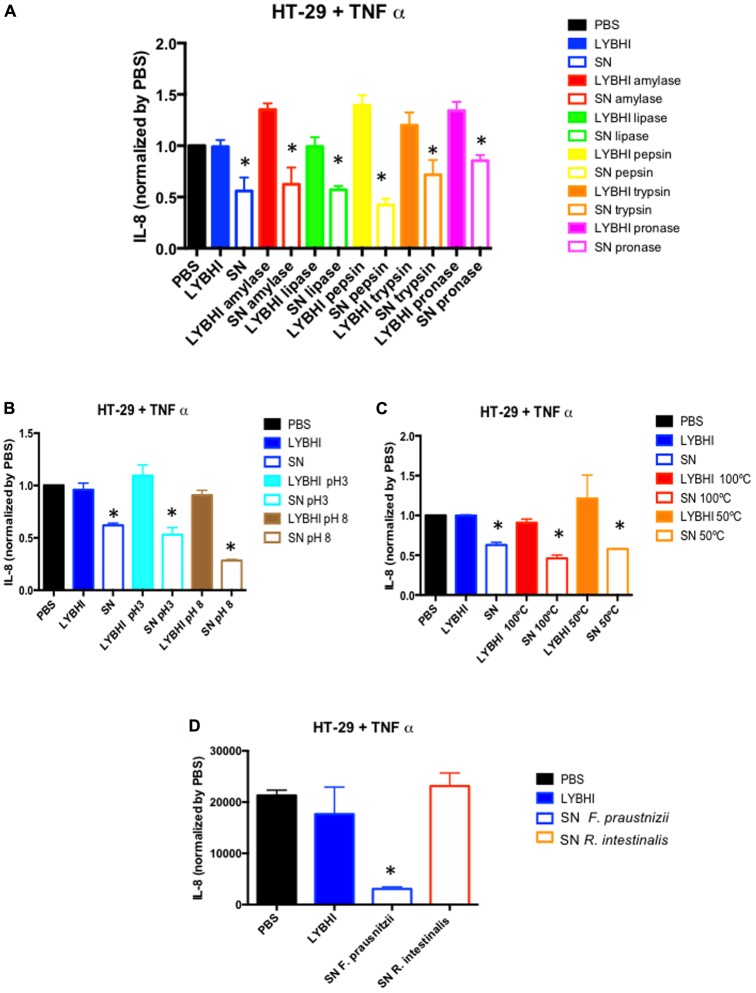
*In vitro*immune-modulatory test on HT-29 cells stimulated with TNF-α. Anti-inflammatory activity of *Faecalibacterium prausnitzii* A2-165 supernatant (SN) and culture medium (LYBHI) submitted to different treatments: **(A)** enzymatic treatments: amylase (red), lipase (green) pepsin (yellow), trypsin (orange) and pronase (pink), **(B)** pH modifications: pH 3 (cyan) and pH 8 (brown), and **(C)** different temperatures: 100°C (red) and 50°C (orange). **(D)** Comparison of *Roseburia intestinalis* DSM14610T SN and *F. prausnitzii* A2-165 SN effect. Bacterial SNs were recovered, filtered and submitted to different treatments (if indicated) before being tested in co-incubation assays with HT-29 cells challenged with TNF-α. IL-8 concentration was determined by ELISA and used as a read-out of the inflammatory status of the cells. The results were normalized by the negative control (PBS) (for material and methods details see Supplementary Data 1). ^∗^*p* < 0.05 *versus* LYBHI (bacterial culture medium) (*n* = 3^∗^3).

Since the first study about *F. prausnitzii* performed in our laboratory almost 10 years ago (Sokol et al., 2008), we have sought to identify the bacterial effectors underlying its beneficial effects. We have been focused on its SN which showed anti-inflammatory properties in both *in vivo* and *in vitro* experiments (Sokol et al., 2008; Martín et al., 2013). Our main hypothesis was that *F. prausnitzii* can produce an anti-inflammatory soluble molecule. First, we tried to identify the chemical nature of the molecule by submitting the SN to several enzymatic and physical methods and as shown in **Figures 1A–C**, none of them were able to suppress the anti-inflammatory “properties” from the SN pointing out the possible presence of more than one effector on *F. praustnizii* SN. Of course, this result can also support an important role of butyrate in *F. prausnitzii* effects, although *in vivo* and *in vitro* experiments point out for a more complex situation (see below). Nevertheless, we need to consider that the beneficial effects of *F. prausnitzii* might not be present only in its SN. For instance, we have found that some beneficial effects, such as the anti-nociceptive one, are observed only when the animals where treated with the bacterium but not with its SN (Miquel et al., 2016).

**FIGURE 2:**
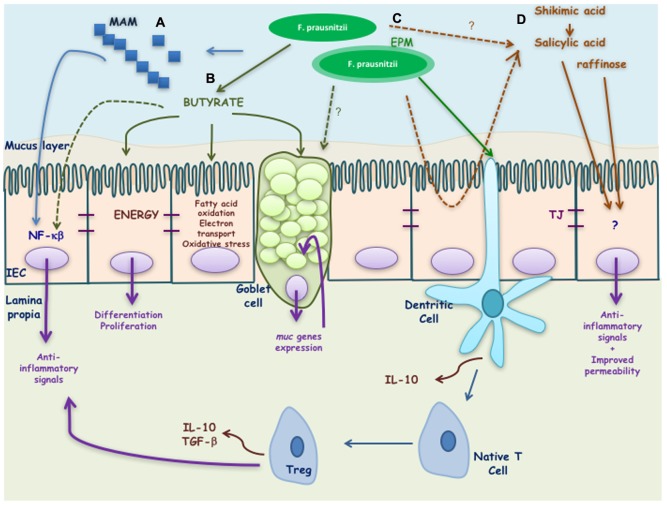
Putative effectors of *Faecalibacterium prausnitzii* and its effects on the host. *F. prausnitzii* exerts its benefic effects by means of different effectors: **(A)** MAM peptides secreted by *F. prausnitzii* block NF-κB activation induced by a pro-inflammatory stimulus. **(B)** Butyrate produced by *F. prausnitzii* inhibits NF-κB activation and interacts with the intestinal epithelial cells (IEC) driving to the activation of different genes involved on the differentiation, proliferation, and restitution of enterocytes. It is also involved on the regulation of fatty acid oxidation, electrons transport chain, oxidative stress, and apoptosis. In goblet cells it has been described to stimulate *muc* genes allowing a high production of mucus. **(C)** EPM produced by *F. prausnitzii* modulates IL-10 cytokine production in antigen presenting cells. Finally, **(D)** salicylic and shikimic acids are anti-inflammatory molecules able to block inflammation induced by a pro-inflammatory stimulus while raffinose is key in maintaining gut permeability.

Some of the putative bacterial effectors identified until now are presented in the **Figure 2** and are described in the next paragraphs.

### Butyrate

Typically, as *F. prausnitzii* is one of the most abundant butyrate-producing species, its beneficial effects have been first attributed to butyrate. Butyrate is a short chain fatty acid (SCFA) well-known for its pleiotropic and beneficial effects in the GIT (Duncan et al., 2009; Macfarlane and Macfarlane, 2011) as well as its immune-modulatory properties *in vitro* (Bocker et al., 2003). Furthermore, it is involved in the cross-feeding between butyrate producer bacteria and *Bifidobacterium* sp. which favors the co-existence of bifidobacterial strains with other bifidobacteria and with butyrate-producing colon bacteria in the human colon with the consequent benefit in the human health (Riviere et al., 2016). Microbial butyrate is considered key for colonic health, as it is an energy source for epithelial cells and is able to modulate oxidative stress and inflammation (Hamer et al., 2008). However, its role remains controversial as its effects seem to be dose- and time-dependent (Martín et al., 2013). In this sense, it also has different effects depending of the cell line tested (Bocker et al., 2003). For instance, regarding cells from intestinal origin, butyrate was found to decrease IL-8 secretion in Caco-2 and human intestinal primary epithelial cells (HIPECs) and to enhance IL-8 production in both HT-29/p and HT-29 MTX cells (Bocker et al., 2003). Furthermore, not all butyrate producing-bacteria have the same anti-inflammatory profile *in vitro.* For instance, in 6 h TNF-α stimulated HT-29 cells, the SN of *Roseburia intestinalis*, a butyrate producer, does not have anti-inflammatory properties while *F. prausnitzii* SN does (**Figure 1D**).

Although the effect of butyrate is clearly present, the anti-inflammatory capacities of *F. prausnitzii* do not seem to be limited to butyrate only. In previous studies, we have shown that *F. prausnitzii-*mediated butyrate production is not the only beneficial bacterial effector linked to this species in colitis models (Sokol et al., 2008; Martín et al., 2013; Miquel et al., 2015b). For instance, we found that the increase of the presence of 4-hydroxybutyric acid in the feces, colon and caecum of dixenic mice colonized with *F. prausnitzii* A2-165 and a strain of *Escherichia coli* compared to monoxenic mice colonized only by the strain of *E. coli* was not directly linked to health parameters in a rat model of acute trinitrobenzenic acid (TNBS)-induced colitis although the increase of production of butyrate has been also verified by gas liquid chromatography (Miquel et al., 2015b). These results support previous findings, where we found that butyrate did not protect mice from TNBS-induced colitis, in contrast to *F. prausnitzii* A2-165 SN (Sokol et al., 2008).

### Other Active Metabolites

The use of a gnotobiotic model including *F. prausnitzii* A2-165 strain and *E. coli* allowed us, using a metabolomic approach, to identify several metabolites that, in contrast to 4-hydroxybutyric acid, are associated to the beneficial effect of *F. prausnitzii* in a TNBS-acute model in rats (Miquel et al., 2015b). These metabolites were the salicylic acid, shikimic acid and raffinose, among others (Miquel et al., 2015b). Salicylic acid is used in the pharmaceutical industry to produce the amine derivate 5-aminosalicylic acid (5-ASA or mesalamine) that it is nowadays used to treat patients suffering from IBD (Messori et al., 1994). Furthermore, shikimic acid is a precursor for the synthesis of several aromatic compounds among which we can also find the salicylic acid through the achorismate synthase pathway (Bochkov et al., 2012). These two compounds point out a key role of *F. praustnizii* in the biosynthesis of salicylic acid, precursor of 5-ASA, both anti-inflammatory molecules that should be linked to the *in vivo* anti-inflammatory effect observed in mice treated with *F. praustnizii*. In contrast, raffinose is an oligosaccharide only fermented by the gut microbiota that is not related to anti-inflammatory effects, but with mucosal permeability, as raffinose permeation is key in maintaining gut permeability (Dawson et al., 1988). The raffinose could thus play a role in the improvement on gut permeability promoted by *F. praustnizii* (Carlsson et al., 2013; Laval et al., 2014). In this sense, we have found that *F. praustnizii* and its SN are able to counterbalance the increase in intestinal barrier permeability in a murine model of gut dysfunction induced by DNBS (Martín et al., 2015). Of note, in this simplified microbiota, we have identified metabolites that can be produced either by the host or by the bacteria, and the direct production of these compounds by *F. prausnitzii* has not yet been proved.

### Microbial Anti-inflammatory Molecule (MAM)

Thanks to a peptidomic analysis using mass spectrometry of *F. prausnitzii* A2-165 strain SN, we have successfully identified seven peptides, all derived from the same anti-inflammatory molecule, a protein of 15 kDa named MAM (ZP05614546.1) (Quevrain et al., 2016). Due to the difficulties to test directly the peptides or the protein, mainly due to solubility problems, indirect strategies were performed to determine their biological effect. Transfection of MAM cDNA in epithelial cells led to a significant decrease in the activation of the nuclear factor (NF)-κB pathway with a dose-dependent effect (Quevrain et al., 2016). This inactivation of NF-κB pathway was also observed *in vivo* using a transgenic model of mice producing luciferase under the control of NF-κB promoter (Breyner et al., 2017). Finally, the administration of a food-grade bacterium, *Lactococcus lactis*, delivering a plasmid encoding MAM was able to alleviate DNBS and DSS induced colitis in mice (Quevrain et al., 2016; Breyner et al., 2017). Although these promising results point out for the strong role of MAM on *F. prausnitzii* SN effect, the persistence of the anti-inflammatory effect of the SN after proteolytic treatment and the ability of *F. prausnitzii* SN to block other inflammatory pathways different from NF-κB (Martin et al., 2014b) point that MAM is not the only bacterial effector mediating *F. praustnizii* SN beneficial effects.

### Extracellular Polymeric Matrix (EPM)

Rossi et al. (2015) found that the biofilm-forming strain *F. praustnizii* HTF-F was able to attenuate the clinical symptoms of DSS-induced colitis in a stronger manner than A2-165 strain. Furthermore, the intra-rectal administration of purified extracellular polymeric matrix (EPM) decrease the disease index of the mice indicating that it contributes strongly to the protective effects of HTF-F strain. However, although the authors concluded that the anti-inflammatory effects of *F. prausnitzii* HTF-F strain may be in part be due to the immune-regulating properties of the EPM, they were not able to rule out other possible strain differences such as colonization ability, stress resistance or *in vivo* fitness, among others. In fact, these parameters might also contribute to the efficacy of the strain as improved survival might impact on butyrate or MAM production for instance.

## Future Perspectives

The lack of clearness about *F. prausnitzii* effectors is linked to the lack of knowledge in its biology and phylogeny. Furthermore, as we mentioned above, probiotic characteristics are strain-specific, and therefore individual studies should be performed in order to determine the individual beneficial effects as well as the individual effectors linked to these effects. If we compare to a classic probiotic group, lactobacilli, we can find that although all lactobacilli are able to produce lactic acid (proved to have beneficial effects in some ecosystems such as the vaginal), not all of them are equipped with the same arsenal of bacterial effectors (bacteriocins, biosulfactants, H_2_O_2_, etc…) that are strain specific. In the case of *Faecalibacterium*, the framework should be similar, as even if all the strains are able to produce butyrate, other possible molecules could be responsible of additional strain-specific beneficial effects.

Recently, we have isolated a collection of novel *F. prausnitzii* strains form healthy volunteers that we have characterized (Miquel et al., 2015a) and analyzed for anti-inflammatory properties with the aim of selecting new NGPs candidates (Martín et al., 2017). The deeper phylogenic analysis of the complete genome of this bacterial collection joint to the genomes already present in the public data bases has revealed that there are at least three separate clusters, spanning the classical Phylogroups I and II already found in *F. praustnizii* (Lopez-Siles et al., 2012, 2016; Martín et al., 2017) and that some strains appear to represent a deeper, more divergent branch of the “*Faecalibacterium prausnitzii*” taxon (Benevides et al., 2017). This new truth about *F. prausnitzii* provides evidence that the phylogeny of *F. praustnizii* should be reconsidered. In line with these results, in the future we should take into account the presence of at least three different genospecies inside *Faecalibacterium* group to better characterize their beneficial effects, their crosstalk with the host and the possible effectors underlying these effects. Furthermore, the pangenome analysis have been performed to get a global view of the genome of this genus. A total of 10,366 core-genome codifying DNA sequences have been found (3.33-fold the average total number of genes of the 17 analyzed strains) (Benevides et al., 2017). Nevertheless, up today no extensive genomic description of this taxon has been finished, being an ongoing task with a key role in the future perspectives of the analysis of this genus.

## Concluding Remarks

In this perspective article, we have tended to highlight the importance and problematic of asserting the probiotic characterization of a NGP, a field on the focus of the research of Frontiers in Microbiology audience. Nowadays, the research community recognizes the importance of *F. prausnitzii* in the human health. A decrease of this bacterium in the GIT has been linked to several diseases and syndromes but it is still not clear if this is a cause or a consequence of them. As mentioned above, this special condition, make this species a unique bacterial sensor and actor in the human health, mainly related with intestinal issues, but not only. Taking advantage of this, its use as NGP is being explored for both human and animal use. However, more research in its phylogeny, physiology, safety, and beneficial effects should be performed to fill the lack that currently exists between the knowledge of the biology of the bacterium and the medical interest that it produces. As an example, the analysis of the possible bacterial effectors taking into account the phylogeny and the biological effects related should be performed in more detail to find the best probiotic candidate and refine its use as biomarker in several human disorders.

## Author Contributions

RM, LB-H, and PL designed the perspective and the experiments. RM wrote the manuscript and performed the experiments. LB-H and PL corrected the manuscript. All the authors approved the last version of the manuscript.

## Conflict of Interest Statement

PL is one of the co-founders of NextBiotiX, a start-up aimed to produce an anti-inflammatory drug based on *Faecalibacterium prausnitzii. The other authors declare that the research was conducted in the absence of any commercial or financial relationships that could be construed as a potential conflict of interest.*
