# Supplementary material for: Searching for the Bacterial Effector: The Example of the Multi-Skilled Commensal Bacterium Faecalibacterium prausnitzii
Source: Front Microbiol. 2018 Mar 6;9:346. doi: 10.3389/fmicb.2018.00346 (PMC5845625; doi:10.3389/fmicb.2018.00346)
Supplement: Supplementary file 1 [file Data_Sheet_1.PDF]

Bacterial strain and growth conditions. *F. prausnitzii* A2–165 (DSM 17677) and *Roseburia intestinalis* (DSM14610T) were grown at 37°C in LYHBHI medium (Brain–heart infusion medium supplemented with 0.5% yeast extract (Difco) and 0,5% L-cysteine (Sigma-Aldrich)) supplemented with cellobiose (1 mg/ml; Sigma–Aldrich), maltose (1 mg/ml; Sigma), and cysteine (0.5 mg/ml; Sigma) in an anaerobic chamber. When necessary the bacteria was grown in a semi-defined medium composed by: KH_2PO_4 (2.62 g/l), $(NH_4)_2SO_4$ (2 g/l), NaCl (2 g/l), $CaCl_2$ (15 mg/l), $MgCl_2$ (150 mg/l) $MnCl_2$ (15 mg/l), $FeCl_3$ (4mg/ml), Vitamin B12 (5mg/l), Vitamin B (Thiamin) (1mg/l), Biotin (1 mg/l), PABA (1 mg/l), Folic acid (1 mg/l), Vitamin K (2 mg/l), 30 mM Acetate, cellobiose (1 mg/ml), maltose (1 mg/ml), glucose (1 mg/l), 2% yeast extract and cysteine (0.5 mg/ml) in an anaerobic chamber.

Supernatant recovery and treatments. The supernatant was recovered by centrifugation of an overnight culture and filter in 0.45 µm filters (VWR). For enzymatic digestion a final concentration of 1mg/ml of protease from *Streptomyces griseus* (pronase), pepsin from porcine gastric mucosa, trypsin from bovine pancreas, lipase or alpha-Amylase (all from Sigma-Aldrich) were added to the supernatant or the medium. After 1H of incubation at 37 ºC the enzymes were inactivated by an incubation of 30 min at 50 ºC.

Eukaryotic cell culture and experiments. HeLa cells (ATCC CCL-2) were grown in Dulbecco's Modified Eagle's minimal essential medium (DMEM) (GibcoBRL) supplemented with 10% (w/v) fetal bovine serum (FBS, GibcoBRL), 2mM L-glutamine (GibcoBRL) and with penicillin G/streptomycin (5000 IU/ml, 5000 µg/ml) (Sigma-Aldrich). Cultures were incubated in 25 cm^2 tissue culture flasks (Nunc) at 37 ºC in a 5% (v/v) CO_2 atmosphere until confluence. For anti-inflammatory assays, 50000 cells per well were seeded in 24-well culture plates (Nunc) and cultivated, with a daily change of the culture medium during 6 days. The assays were performed seven days after seeding. Twenty-four hours before bacterial challenge, the culture medium was changed for a medium with 5% FBS. The bacterial supernatant at 20% , the control medium, PBS (GibcoBRL), the different chromatography fractions or the molecules to test were added and the cells were stimulated simultaneously with human recombinant TNF-alpha (5 mg/ml; Sigma–Aldrich) in DMEM medium with 5% FBS for 6 h. After incubation, cell supernatants were collected and frozen at 80°C with a cocktail of

protease inhibitors EDTA-free (Roche) until analysis. The IL-8 level was determined in cell supernatants using Human IL-8 ELISA kit (Biolegend) according to the manufacturer's recommendations.
